# Natural Killer (NK) Cells in Tumor Immunity: Limitations and Therapeutic Potential with a Focus on Nasopharyngeal Carcinoma and Comparison with T-Cell-Based Therapies

**DOI:** 10.3390/cells15100913

**Published:** 2026-05-15

**Authors:** Anna Makowska, Udo Kontny

**Affiliations:** Division of Pediatric Hematology, Oncology and Stem Cell Transplantation, RWTH Aachen University, Pauwelsstraße 30, D-52074 Aachen, Germany; ukontny@ukaachen.de

**Keywords:** nasopharyngeal carcinoma, NPC, tumor immunity, natural killer cells

## Abstract

Natural killer (NK) cells are increasingly recognized as a complementary platform to T-cell-based cancer immunotherapies. Their innate, MHC-unrestricted recognition, capacity to mediate antibody-dependent cellular cytotoxicity (ADCC) and comparatively favorable toxicity profile have given rise to a broad therapeutic pipeline that includes cytokine-supported regimens, adoptive NK products, bispecific and trispecific NK engagers, and chimeric antigen receptor (CAR)-engineered NK cells. Clinical data, particularly in hematologic malignancies, show that NK-cell-based strategies can be safe and biologically active, although limited persistence, suboptimal trafficking and immune escape remain key challenges. Nasopharyngeal carcinoma (NPC), an Epstein–Barr virus (EBV)-driven epithelial cancer, illustrates how a tumor microenvironment (TME) can simultaneously impair NK function and create specific vulnerabilities that NK-focused therapies can exploit. This review summarizes NK biology and current therapeutic platforms, analyzes major limitations, highlights the specific context of NK-cell-based strategies in NPC and compares NK- and T-cell-based therapies with an emphasis on clinical translation.

## 1. Introduction—Biology of NK Cells and Their Role in Tumor Immunosurveillance

NK cells are cytotoxic innate lymphocytes that integrate signals from germline-encoded activating and inhibitory receptors, including the natural cytotoxicity receptors and the Fc receptor CD16, which mediates ADCC against antibody-coated targets (Table 1) [1,2,3]. Inhibitory receptors on NK cells—most prominently KIRs and NKG2A/CD94—sense self MHC class I and transmit a dominant “do not kill” signal. When tumor cells lose or fail to stabilize MHC I (“missing self”), that inhibitory brake is removed, and NK cells more readily degranulate and lyse the target [4,5]. This is classically demonstrated in murine β2-microglobulin-deficient systems [4,5,6,7,8,9,10], which show a global failure of MHC I surface expression, and in the RMA-S tumor model, a mutant mouse T-cell lymphoma cell line derived from RBL-5 by ethylmethane sulfonate mutagenesis and anti-H-2 antibody/complement selection [4,6]. Because RMA-S has defective peptide loading, it expresses low and unstable levels of surface murine MHC I, making it highly susceptible to NK cell-mediated killing. Accordingly, RMA-S lymphoma is widely used to model tumor–NK interactions driven by MHC I loss and to test immunomodulatory strategies (e.g., checkpoint pathways, vaccination approaches) in a setting where NK sensitivity is mechanistically linked to impaired MHC I presentation [1,2,4,5,6,7]. However, this is a murine model, and extrapolation to human tumors requires caution [4,5,6,7]. Activating receptors such as NKG2D, DNAM-1 and NKp30/NKp44/NKp46 detect stress-induced ligands (e.g., MICA/B, ULBPs, PVR/NECTIN2) upregulated on virus-infected and malignant cells and drive NK-mediated cytotoxicity in vitro and in vivo [1,2,3]. In addition, NK cells constitutively express SLAMF7 (CS1/CRAAC), a self-ligand activating receptor highly expressed on plasma cells and leveraged therapeutically by the humanized antibody Elotuzumab (Empliciti) in multiple myeloma, as well as in SLAMF7-directed CAR NK approaches with limited on-target, off-tumor toxicity [11,12,13,14].

CD16 (FcγRIIIa) enables NK-cell ADCC against IgG1-opsonized targets and is a major effector pathway for antibodies such as rituximab and trastuzumab; NK-cell depletion or CD16 blockade markedly reduces their antitumor activity in preclinical models [1,2].

At the effector level, NK cells eliminate targets primarily through perforin/granzyme-dependent cytotoxicity and death receptor pathways mediated by tumor necrosis factor-related apoptosis-inducing ligand (TRAIL) and Fas ligand (FasL). Upon activation, NK cells polarize cytotoxic granules toward the immunological synapse and release perforin together with granzymes, particularly granzyme B; perforin forms pores in the target-cell membrane that allow granzymes to enter and trigger caspase-dependent and caspase-independent apoptosis. Gene-targeted mice lacking perforin show profoundly impaired NK cell-dependent tumor control, with increased incidence and earlier onset of spontaneous lymphomas and enhanced growth and metastasis of carcinogen-induced or transplantable tumors compared with wild-type controls, directly linking perforin to NK cell-mediated tumor immunosurveillance [1,2,3,15,16,17]. These mechanistic links derive predominantly from mouse models. Granzyme B expression in NK cells is likewise critical for efficient killing of a broad range of tumor targets, and high densities of intratumoral NK cells that co-express perforin and granzyme B correlate with improved prognosis in several human solid tumors [2,18]. In parallel, activated NK cells express TRAIL and FasL, which engage TRAIL receptors and Fas (CD95) on susceptible tumor cells. This interaction induces apoptosis via death-inducing signaling complexes. FasL expression and Fas-mediated killing by NK cells were demonstrated in primary mechanistic studies [17,19]. Evidence from murine tumor models further implicated TRAIL in NK-cell-mediated control of tumor metastasis and IFN-γ-dependent tumor immunosurveillance. TRAIL neutralization reduced NK-mediated antimetastatic activity and enhanced tumor development in these mouse models [1,2,3,20,21,22]. Conversely, many tumors downregulate Fas, alter TRAIL receptor expression or upregulate decoy receptors, illustrating that evasion death receptor-mediated cytotoxicity is an important component of NK escape in cancer [23].

Functional depletion or genetic impairment of NK cells in mice—using antibodies such as anti-asialo GM1 or NKp46-DTR models—consistently accelerates tumor growth and metastasis in carcinogen-induced, spontaneous and transplantable tumor systems, whereas intact NK compartments delay or prevent tumor outgrowth [1,2]. These loss-of-function data also derive predominantly from mouse models. In patients, higher intratumoral NK-cell density has been associated with improved survival in specific tumor types, including colorectal carcinoma, gastric carcinoma, lung adenocarcinoma and squamous cell lung cancer [24,25,26], supporting a protective role of NK-cell infiltration in at least some human cancers [2,18].

**Figure 1:**
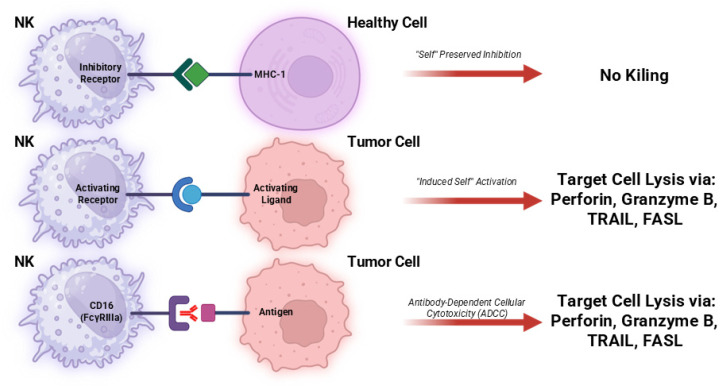
Recognition of target cells and NK cell-mediated lysis. NK cells identify and eliminate abnormal cells through a balance of inhibitory and activating signals. Healthy cells: inhibitory NK cell receptors bind to MHC class I molecules, suppressing NK cell activation and preventing lysis. Tumor cells: reduced MHC class I expression weakens inhibitory signaling, allowing activating receptors to trigger NK cell cytotoxicity. This involves enzymes: perforin and granzymes, ligands: FasL and TRAIL, cytokines. Antibody-coated cells: The CD16 activating receptor binds to antibodies attached to the target cell, inducing lysis via antibody-dependent cellular cytotoxicity (ADCC). Created in Biorender. Anna Makowska. (2026).

Single-cell transcriptomic and pan-cancer profiling studies show that tumor-associated NK cells often acquire an “exhausted” phenotype. This phenotype is characterized by upregulation of inhibitory receptors (e.g., NKG2A, TIGIT, PD-1), metabolic stress signatures and reduced expression of cytotoxic effector gene [27,28]. These changes are particularly evident in advanced disease. In this setting, exhausted NK-cell subsets are associated with poor clinical outcome (Figure 1) [7,18]. In addition, LLT1 (CLEC2D) is an inhibitory tumor-escape ligand expressed on glioblastoma, prostate cancer, TNBC, squamous cell carcinoma and colorectal carcinoma. It binds NKR-P1A/CD161 (KLRB1) on NK cells [29,30,31,32].

## 2. NK-Cell-Based Cancer Therapies

### 2.1. Cytokine-Based Activation of Endogenous NK Cells

High-dose interleukin-2 (IL-2) was the first strategy used to activate and expand endogenous NK cells. It can induce durable responses in a small subset of patients with metastatic melanoma or renal cell carcinoma. However, IL-2 also expands FOXP3^+^ Tregs. It can also cause substantial toxicity, including vascular-leakage syndrome. These limitations restrict its use to selected patients [1,2,3,33,34,35].

Interleukin-15 (IL-15)-based agonists were developed to stimulate NK and memory CD8 T cells without directly feeding CD25high Tregs [3,35]. The IL-15 superagonist N-803 (ALT-803) induces marked proliferation and activation of NK- and CD8 T cells with limited effects on Tregs and has shown objective responses in hematologic malignancies and non-muscle-invasive bladder cancer, where intravesical ALT-803 plus BCG led to high complete-response rates and regulatory approval [36,37]. Other IL-15 agonists, such as nanrilkefusp alfa (SOT101), expand NK/CD8 compartments but induce mainly stable disease as monotherapy in solid tumors [38]. Overall, IL-2/IL-15–family cytokines can robustly expand and activate endogenous NK cells and occasionally drive tumor regression, but durable control is uncommon, and current efforts focus on combinations with checkpoint inhibitors, antibodies or BCG rather than cytokine monotherapy (Table 2) [1,2,3,33,36,37,38,39].

### 2.2. Antibody-Mediated ADCC and NK Engagers

ADCC via FcγRIIIa/CD16a on NK cells is an important effector mechanism for IgG1 antibodies such as rituximab, trastuzumab and cetuximab. However, it is not the sole Fc-dependent pathway in vivo. Cells of the mononuclear phagocyte system, particularly monocytes and macrophages, contribute through FcγR-mediated antibody-dependent cellular phagocytosis and related effector mechanisms, while other FcγR-expressing myeloid cells, including neutrophils, may also participate depending on the antibody, tumor type and tissue context. Clinical data in B-cell lymphoma indicate that patients carrying the high-affinity FCGR3A-158V/V genotype have better responses to rituximab-based regimens. This association is particularly evident in follicular lymphoma treated with rituximab-dominant regimens. Similar associations between activating FcγR polymorphisms and clinical outcome have also been reported in some trastuzumab-treated HER2-positive breast cancer cohorts. However, these genotype–response associations are not uniform across all chemoimmunotherapy settings. FcγR-deficient mouse models demonstrate that FcγR signaling is required for rituximab efficacy. Complementary in vivo studies further support an important role for mononuclear phagocytes, particularly macrophages, in CD20 antibody-mediated clearance [46,47,48,49,50,51,52].

Fc-engineered antibodies such as obinutuzumab (GA101) increase CD16a affinity through Fc defucosylation and mediate stronger NK-cell ADCC, translating into improved outcomes in CLL and follicular lymphoma [53,54,55,56]. CD16a is also dynamically regulated in human NK cells by activation-induced ADAM17-mediated shedding, which reduces surface CD16a after NK-cell activation or engagement with antibody-coated target cells. In human in vitro systems, pharmacologic ADAM17 inhibition preserves CD16/CD16a expression and enhances CD16-mediated effector functions, including ADCC by expanded NK cells against trastuzumab-treated human breast cancer cell lines [57,58].

Combining tumor-targeting antibodies with NK-activating cytokines can further enhance FcγRIIIa/CD16a-dependent NK-cell responses. In human in vitro studies, N-803 increases rituximab-triggered NK-cell degranulation, IFN-γ production and ADCC against human B-cell lymphoma targets. In vivo, enhanced lymphoma clearance has been demonstrated in immunodeficient mouse xenograft models, including models using adoptively transferred human NK cells. Similar cytokine-mediated potentiation of antibody-dependent effector functions has been observed with other tumor-targeting antibodies in murine tumor models and human in vitro carcinoma-cell systems [59,60,61]. Engineered NK-cell products with high-affinity, non-cleavable CD16a (hnCD16), which combines the high-affinity 158V variant with resistance to ADAM17-mediated cleavage, as well as triple-edited “iADAPT” induced pluripotent stem cell (iPSC)-derived NK cells expressing hnCD16 and membrane-bound IL-15/IL-15Rα, show improved and durable ADCC against multiple antibody-coated tumor targets [62,63,64]. Analogous genetic engineering of primary human peripheral-blood NK cells by CRISPR/Cas9-mediated ADAM17 knockout prevents activation-induced CD16a shedding and augments antibody-triggered NK-cell functions in vitro, including rituximab-mediated ADCC against human Raji lymphoma cells [65].

Bispecific and trispecific NK-cell engagers directly link NK cells to tumor antigens. In IL-15-containing TriKE molecules, the cytokine moiety is incorporated into the engager molecule and can provide localized IL-15 signaling to the recruited NK cell at the NK-cell–tumor-cell interface. AFM13, a CD30/CD16A TandAb, is well tolerated and shows clinical activity in Hodgkin lymphoma, with further improvement when pre-complexed with cord blood-derived NK cells [66,67]. The CD16/IL-15/CD33 TriKE enhances NK-cell expansion and antitumor activity against CD33^+^ acute myeloid leukemia (AML) and can also rescue dysfunctional NK cells from myeloid-derived suppressor cell (MDSC)-mediated inhibition. In vitro, studies using primary samples from patients with MDS and AML showed that this TriKE increases NK-cell cytotoxicity, degranulation, and cytokine production against CD33^+^ targets. It also effectively eliminates MDSCs, thereby reversing MDSC-mediated suppression of NK-cell function. In vivo, functional evaluation in NSG xenograft mouse models engrafted with human AML (HL-60) cells and human NK cells demonstrated reduced tumor burden, enhanced NK-cell expansion, and improved persistence. Clinically, early-phase studies have evaluated related next-generation TriKEs, including GTB-3550 and GTB-3650. These agents have shown acceptable safety in patients with high-risk MDS and relapsed/refractory AML. They also induce substantial NK-cell activation and proliferation. These findings support their continued development [68,69,70]. These platforms converge on NK ADCC as a central axis in antibody-based immunotherapy, although optimal dosing and durability remain to be defined.

Subsequent translational data add nuance to this picture. AFM13 monotherapy in relapsed/refractory classical Hodgkin lymphoma showed manageable safety. However, single-agent durability was limited. In contrast, AFM13 pre-complexed with cord blood-derived NK cells produced substantially higher activity in phase 1 CD30+ lymphoma studies. These findings support combination-based rather than single-agent development [71,72]. In solid tumors, first-in-human AFM24 monotherapy established a recommended phase 2 dose of 480 mg but yielded mainly disease stabilization, indicating that combination regimens are likely the more informative translational setting [73].

### 2.3. Adoptive Transfer of NK Cells

Adoptive NK-cell therapy uses NK cells derived from peripheral blood, umbilical cord blood, iPSCs or cell line NK-92. Haploidentical NK cells from related donors are commonly infused after lymphodepleting chemotherapy to promote NK-cell expansion, persistence and antitumor activity. In AML, they have shown feasibility, safety and antileukemic activity. Responses correlate with in vivo expansion and KIR–HLA ligand mismatch and minimal GVHD (graft-versus-host disease—when donor immune cells attack recipient tissues, classically involving the skin, liver, and gastrointestinal trac) [74,75,76,77].

Cytokine-induced memory-like (CIML) NK cells are generated by brief ex vivo pre-activation of human NK cells with IL-12, IL-15 and IL-18. These cells display enhanced effector function, including increased IFN-γ production and antileukemic activity. In a phase I study in relapsed/refractory AML, donor-derived CIML NK cells expanded in vivo and trafficked to the bone marrow. Clinical responses were observed in five of nine evaluable patients, including complete remissions in four patients, without major treatment-associated CRS, neurotoxicity or GVHD [54,55,56,57,58].

More recent first-in-human data also extend the CIML NK concept beyond AML: in advanced head and neck cancer, the adoptive transfer of CIML NK cells combined with the IL-15 superagonist N-803 and ipilimumab was feasible, induced a proliferative NK-cell state and showed biologic activity, although efficacy remains preliminary [53].

Off-the-shelf NK-cell products include iPSC-derived platforms such as FT500, which have shown favorable safety, feasibility of repeated dosing, and preliminary disease stabilization in patients with advanced cancers. More broadly, multiplex-engineered iPSC-derived NK cells—incorporating chimeric antigen receptors (CARs), high-affinity non-cleavable CD16 (hnCD16), and IL-15/IL-15Rα fusion constructs—are being developed as standardized allogeneic therapies, with a primary focus on hematologic malignancies and expanding application in solid tumors. Among blood cancers, major targets include acute myeloid leukemia (AML), B-cell malignancies such as B-cell acute lymphoblastic leukemia (B-ALL), chronic lymphocytic leukemia (CLL), and B-cell non-Hodgkin lymphoma (B-NHL), as well as multiple myeloma and other lymphomas. Representative examples include FT538, which is being explored in relapsed/refractory AML and multiple myeloma, and FT596, which targets CD19-positive B-cell malignancies, including B-ALL and B-NHL. In parallel, these platforms are increasingly being adapted for solid tumors, including pancreatic cancer, ovarian cancer, glioblastoma, lung cancer, and breast cancer, particularly triple-negative breast cancer, through CARs directed against antigens such as CD44v6, TROP2, or MUC1. Functionally, hnCD16 enhances antibody-dependent cellular cytotoxicity and supports combination strategies with therapeutic monoclonal antibodies across multiple tumor types, whereas IL-15/IL-15Rα fusion constructs promote in vivo persistence and expansion without exogenous cytokine support. Additional engineering features, such as CD38 knockout, further tailor these products for specific indications, for example by preventing fratricide during daratumumab-based treatment of multiple myeloma. Together, these data position engineered iPSC-derived NK cells as a versatile off-the-shelf immunotherapy platform spanning both hematologic and solid malignancies [78,79,80]. Multiplex-engineered iPSC-derived NK cells with CARs, high-affinity CD16 and IL-15/IL-15Rα fusions are being developed as standardized, allogeneic cell therapeutics [78,79,80].

Among iPSC-derived NK-cell products, FT596 is the most clinically advanced. In a first-in-human phase 1 study in relapsed/refractory B-cell lymphoma, FT596 showed favorable tolerability, outpatient feasibility and durable responses, including activity after prior CAR-T exposure, particularly in rituximab-containing regimens [81]. By contrast, the BCMA-targeted iPSC-derived CAR-NK product FT576 is less clinically advanced than FT596. Early clinical data suggest encouraging safety and preliminary activity, both as monotherapy and in combination with daratumumab, but mature peer-reviewed efficacy data remain limited [82,83].

The NK-92 cell line, an IL-2-dependent human NK-cell line established in 1992 from the peripheral blood of a patient with non-Hodgkin lymphoma, offers virtually unlimited ex vivo expansion and enables the generation of homogeneous GMP-grade cell products. Irradiated NK-92 has been safely administered in phase I trials in solid and hematologic malignancies, with mild infusion reactions, no GVHD and evidence of transient tumor control [84,85,86]. NK-92 now serves as a backbone for multiple CAR-NK-92 products targeting CD19, HER2, EGFR and others, which show strong preclinical activity and are in early clinical evaluation [86,87]. Collectively, these data illustrate the versatility of adoptive NK-cell transfer and highlight the attractiveness of allogeneic NK products, with negligible GVHD risk, for scalable off-the-shelf manufacturing and repeated dosing.

### 2.4. CAR-NK Cells

CAR-NK cells combine NK cells’ innate recognition and allogeneic safety with CAR-based antigen specificity. Cord blood-derived CD19 CAR-NK cells co-expressing IL-15 and inducible caspase-9 have shown encouraging results: in an initial trial, 8/11 patients with relapsed/refractory CD19^+^ lymphoid malignancies responded, including seven complete remissions, with CAR-NK persistence up to 12 months and no CRS, neurotoxicity, or GVHD [88]. An expansion cohort confirmed antitumor activity and low toxicity in a larger population [89], consistent with preclinical data showing long-term persistence and potent anti-CD19 activity of IL-15-armored CAR-NK cells [90].

In preclinical in vivo studies, BCMA-targeted CAR-NK cells (B-Cell maturation antigen-targeted chimeric antigen receptor natural killer cells) armed with IL-15 have shown enhanced antitumor efficacy in mouse xenograft models of multiple myeloma. In these immunodeficient mice engrafted with human myeloma cells, IL-15 co-expression improved NK-cell persistence and functional activity, resulting in superior control of tumor growth compared with non–IL-15-armored CAR-NK cells. Encouraged by these findings, BCMA-targeted CAR-NK therapies have advanced into early-phase clinical testing in patients with relapsed/refractory multiple myeloma. This strategy exploits BCMA, a TNF receptor superfamily member expressed predominantly on plasmablasts and plasma cells. BCMA binds the ligands BAFF (B-cell activating factor, also known as TNFSF13B) and APRIL (a proliferation-inducing ligand, TNFSF13), which are key survival factors in the bone marrow microenvironment that promote plasma-cell maintenance and, in multiple myeloma, can support malignant plasma-cell survival. Thus, targeting BCMA is biologically attractive because BCMA is preferentially expressed on plasma cells, including malignant plasma cells in multiple myeloma, and marks a cell population that depends on BAFF/APRIL-mediated survival cues within the bone marrow microenvironment [91,92]. Additional early studies with CAR-NK products targeting MUC1 or NKG2D ligands report partial responses or stable disease with minimal off-tumor toxicity [93].

Across programs, CAR-NK cells show a lower incidence of CRS. Across clinical programs, CAR-NK cells appear to be associated with a lower incidence of CRS, ICANS and GVHD than CAR-T cells. ICANS, or immune effector cell-associated neurotoxicity syndrome, is a neurologic toxicity observed after cellular immunotherapies, particularly CAR-T-cell therapy, and can manifest as confusion, aphasia, tremor, seizures or decreased consciousness. CAR-NK cells can be generated from multiple allogeneic sources. These include PBMCs, cord blood, iPSCs and NK-92 cells. They are also well suited for banked manufacturing. This may enable broader and less costly access than individualized CAR-T therapy [90,93,94]. However, persistence is often limited outside IL-15-armored platforms, especially for irradiated NK-92, and responses in solid tumors are generally modest and short-lived. Immunosuppressive TMEs with hypoxia, TGF-β and checkpoint ligands further impair trafficking and function [90,94,95,96,97], motivating engineering of CAR-NK cells with cytokine armoring, chemokine receptors, checkpoint disruption and metabolic enhancements, and their integration into rational combinations with antibodies, cytokines and checkpoint inhibitors [91,94,95,96,97,98,99,100].

Not all programs progressed despite acceptable safety. NKX101, an allogeneic, off-the-shelf CAR-NK cell product targeting NKG2D ligands and engineered to express membrane-bound IL-15, initially showed complete remissions in a small AML cohort. However, broader interim phase 1 data showed a lower overall complete remission/complete remission with incomplete hematologic recovery rate across the expanded patient population, after which enrollment was closed and the program was deprioritized. This illustrates the translational attrition still faced by allogeneic CAR-NK platforms [101].

## 3. Barriers to NK-Cell Therapy in Solid Tumors

### 3.1. Immunosuppressive Tumor Microenvironment

In solid tumors, NK cells face physical and biochemical barriers that impair trafficking and function. Dense, collagen-rich extracellular matrix and abnormal vasculature form stromal barriers around tumor nests, increasing interstitial pressure and reducing pore size. As a result, NK cells often accumulate in perivascular or stromal regions but are excluded from tumor-cell nests embedded in desmoplastic, collagen-rich stroma [102,103,104,105].

TME-derived soluble factors, including TGF-β, IL-10, and PGE_2_, suppress NK cells by downregulating activating receptors, reprogramming metabolism, and blunting IL-15-driven proliferation and IFN-γ production, thereby promoting a quiescent state with reduced cytotoxicity [106,107,108,109]. In many solid tumors, including pancreatic, ovarian, and lung cancers as well as glioblastoma, hypoxia together with the buildup of lactate and adenosine creates a metabolically hostile microenvironment that further impairs NK-cell metabolism and function, reducing degranulation and IFN-γ production [109,110,111,112]. Hypoxic niches limit the fitness and persistence of adoptively transferred NK cells, although forced oxidative metabolism can partially restore effector function [113].

Chronic receptor engagement also drives NK exhaustion. Persistent exposure to NKG2D ligands uncouples NKG2D signaling from cytotoxicity [114,115]. Tumors upregulate HLA-E and ligands for inhibitory receptors such as NKG2A, while NK cells acquire high expression of NKG2A, TIGIT, TIM-3 and PD-1, with reduced degranulation and cytokine production and transcriptional and metabolic signatures of exhaustion; abundance of these subsets correlates with poor prognosis in several cancers [27,116,117,118]. These barriers help explain why NK-based therapies are more successful in hematologic malignancies than in solid tumors unless combined with TME-remodeling strategies [3,4,7,27,69,106,108].

### 3.2. Limited Persistence and Expansion

Adoptively transferred NK cells usually expand transiently and persist only briefly, limiting response depth and durability. In haploidentical NK trials in AML, donor NK cells were often undetectable beyond a few weeks, and complete remissions correlated with robust NK expansion [74]. Similar patterns are seen with CIML NK and unmodified allogeneic NK products, where proliferation wanes without continued cytokine or antigenic stimulation [54,55,56,108]. NK-92-based therapies must be irradiated before infusion, eliminating proliferation and restricting activity to a short window [84,85,86].

Accordingly, current engineering strategies aim to overcome limited NK-cell persistence and trafficking by incorporating membrane-bound IL-15, which supports NK-cell survival and effector function, and by adding chemokine receptors such as CXCR1 or CXCR2 to improve migration toward IL-8- or CXCL1/2-rich solid tumors [119,120,121,122,123].

IL-15 armoring, via IL-15/IL-15Rα fusion molecules or IL-15-containing CARs, improves NK survival, proliferation and long-term antitumor activity in preclinical models and has translated into prolonged CAR-NK persistence in patients [88,124,125,126,127]. However, systemic IL-15 exposure is associated with inflammatory toxicities (fever, hypotension, liver enzyme elevations, capillary leak) in IL-15 and IL-15 superagonist trials [59,60,61,128]. Next-generation CAR-NK designs therefore use restricted IL-15 expression and suicide switches (e.g., inducible caspase-9) and careful clinical monitoring to balance persistence against potential systemic toxicity [1,3,7,37,63,124].

### 3.3. Antigen Heterogeneity and Escape

Single-antigen targeting by CAR-T or CAR-NK cells creates strong selective pressure for antigen-loss variants. CD19-negative relapses after CD19 CAR-T, driven by CD19 downregulation, alternative splicing or lineage switch, are a well-documented example [128,129,130,131,132]. Similar reductions in HER2 density or altered receptor biology contribute to resistance to HER2-targeted therapies [133,134,135].

To counter this, multi-target CAR-NK constructs recognizing two or more antigens (e.g., dual/tandem CARs) have shown more durable tumor control and delayed antigen-negative relapse in preclinical models [63,136]. NKG2D- or DNAM-1-based CARs broaden recognition to sets of stress-induced ligands such as MICA/B, ULBPs, PVR/CD155 and NECTIN-2/CD112, which are frequently co-expressed across malignancies, thereby buffering against loss of any single ligand [77,136]. DNAM-1 CAR-NK-92 cells show strong activity against diverse ligand-positive tumors, and their efficacy can be enhanced in vitro by Nutlin-3a, an inhibitor of MDM2, a negative regulator of the tumor suppressor p53. By restoring p53 activity, Nutlin-3a can increase tumor-cell expression of DNAM-1/NKG2D ligands and thereby sensitize tumor cells to DNAM-1-engineered NK-cell killing [3,49,134,135,136,137,138].

Combining CAR-NK cells with BiKEs, TriKEs or therapeutic antibodies allows NK cells to use both CAR-mediated and CD16-mediated recognition simultaneously, reducing the likelihood of single-antigen escape [34,35,76,89,90,91]. Clinical proof that such strategies will be of benefit is still emerging.

### 3.4. Manufacturing and Standardization

NK-cell manufacturing is more demanding than for T cells. Clinical-scale expansion from PBMCs often uses feeder cells (e.g., K562-mbIL21-41BBL) or complex cytokine cocktails, with considerable donor-to-donor and batch variability in yield, phenotype and receptor expression [139,140]. Slight differences in cytokine dosing or culture conditions can alter the CD56^bright^/CD56^dim^ balance and exhaustion marker expression, complicating cross-trial comparison [140]. Fresh NK cells generally outperform cryopreserved and thawed cells, which often require rest and cytokine re-stimulation to recover full function [141].

Source heterogeneity adds to complexity. PBMC-, cord blood-, iPSC- and NK-92-derived NK products differ in scalability, phenotype and safety. PBMC-derived NK cells are physiologic but donor-dependent; cord blood NK cells are bankable but require robust expansion; NK-92 is homogenous and easily expanded but must be irradiated, preventing in vivo proliferation [35,142,143]. iPSC-derived NK platforms (e.g., FT500, FT516) provide large, standardized lots but require stringent control of differentiation, genomic stability and potency assays [144,145,146].

This diversity makes it challenging to define class-wide potency assays and identity markers for “NK therapies” and complicates regulatory approval [7,147,148,149,150]. Ongoing efforts aim to harmonize minimal characterization panels (e.g., CD56/CD3, KIR repertoire, CD16, NKG2A/NKG2D), reference functional assays and manufacturing reporting standards to enable meaningful comparison between NK-cell products [7,145,150,151].

### 3.5. Safety Considerations

On-target/off-tumor toxicity is a shared concern for CAR-T and CAR-NK therapies. Fatal pulmonary toxicity after high-affinity HER2 CAR-T in a patient with metastatic colon cancer, attributed to low-level HER2 expression on lung tissues, illustrates the risk of targeting antigens that are not strictly tumor-specific [104]. Although similar catastrophic events have not been observed with HER2 CAR-NK cells, preclinical work shows that CAR-NK cells can lyse normal cells engineered to express target antigens, underscoring the need for rigorous expression profiling and careful dose escalation [7,148,152]. NKG2D- or DNAM-1-based CARs carry the added risk of targeting non-malignant cells that upregulate stress ligands under inflammation [153,154].

Cytokine-mediated toxicity is another consideration for heavily armored NK-cell products. Here, IL-15 armoring generally denotes genetic engineering of CAR-NK cells to express IL-15 or an IL-15-containing construct, rather than transient ex vivo exposure to IL-15 before infusion. This distinction is important, because engineered IL-15 expression may sustain autocrine or local paracrine signaling in vivo. Although IL-15-armored CAR-NK cells have been well tolerated to date, the inflammatory toxicity observed with recombinant IL-15 and N-803 suggests that potent IL-15 signaling remains a potential liability and warrants careful control in highly IL-15-driven cell products [36,88,91,155]. Similar issues are anticipated for IL-12- or IFN-α-armored NK cells if cytokine release is not spatially restricted [149,154].

Finally, multiplex-edited, long-lived allogeneic NK products carry theoretical long-term risks such as insertional mutagenesis and malignant transformation [37,63,149,155]. Consequently, many next-generation CAR-NK constructs include suicide switches (e.g., inducible caspase-9) and are subject to prolonged post-infusion surveillance, like gene therapy and CAR-T products [63,88,91,150,156].

## 4. Nasopharyngeal Carcinoma as a Model for NK-Cell Therapy

Nasopharyngeal carcinoma (NPC) is an epithelial malignancy with highest incidence in Southern China and Southeast Asia, where non-keratinizing, EBV-positive NPC predominates, and is relatively rare in Western populations [157,158,159]. Chronic latent EBV infection reprograms the TME into an immunosuppressive niche with abundant stroma, myeloid reprogramming and high expression of immune checkpoints and inhibitory cytokines [157,160].

EBV-positive NPC lesions are among the most immune-infiltrated epithelial tumors, with dense T-, B-, myeloid and NK-cell infiltrates in stroma and peritumoral regions, making NPC a useful model of virus-driven immune sculpting rather than simple immune exclusion [157,159,160].

Single-cell RNA-seq studies show that cytotoxic lymphocytes, including CD57^+^/CD56^+^ NK cells, are enriched in stromal regions but under-represented in malignant epithelial nests, indicating partial immune exclusion within an otherwise inflamed TME—i.e., a microenvironment with abundant immune infiltration and pro-inflammatory signaling overall [161,162,163,164]. Spatial and ligand–receptor analyses reveal strong interferon signaling plus abundant inhibitory interactions. NPC biopsies display mosaics of immune-excluded nests surrounded by fibroblast-rich stroma and intensely inflamed regions, defining distinct patterns of immune escape within the same lesion [162,163,164,165].

An NK-focused multi-omics study in non-keratinizing NPC found global downregulation of proteins linked to NK cytotoxicity. These proteins included lymphocyte-specific protein tyrosine kinase, protein kinase C beta type, protein tyrosine phosphatase non-receptor type 11, integrin alpha L (CD11a), caspase-3 and mitogen-activated protein kinase 3 (ERK1). These changes were observed compared with normal mucosa. They were accompanied by reduced CD56^+^ NK-cell numbers and lower granzyme B expression [166]. Single-cell analysis identified tissue-resident NK subsets (ZNF683high) with exhaustion signatures, high TIGIT/LAG-3 expression and suppressed cytotoxic pathways [167]. Ligand–receptor analysis implicated LGALS9–CD44/CD45/HAVCR2 interactions between mast cells and NK cells in NK dysfunction [166]. Overall, intratumoral NK cells in NPC are numerically reduced, skewed toward tissue-resident, checkpoint-high exhausted phenotypes and distributed in a patchwork of excluded and inflamed regions [157,158,159,165,166,167,168], highlighting them as both targets and victims of EBV-driven immune remodeling.

## 5. EBV-Driven NK-Cell Evasion Mechanisms in NPC

EBV shapes NK-cell evasion in NPC by modulating tumor-intrinsic and microenvironmental pathways. EBV-positive NPC cell lines and tumors exhibit increased B7-H3 (CD276) expression compared with adjacent mucosa, and high B7-H3 correlates with reduced NK infiltration and poor prognosis [4,169]. In preclinical EBV^+^ NPC xenograft mouse models, suppression of tumor-cell B7-H3 helped restore NK-cell effector function and improved in vivo tumor control, implicating B7-H3 as a relevant mediator of NK-cell inhibition in this setting [163,164].

NPC cells also manipulate stress ligands and MHC. Malignant epithelial cells can downregulate classical HLA class I in parts of the lesion. They can also show variable expression of NKG2D ligands, including MICA/B and ULBPs. In addition, MICA/B can be shed or packaged into exosomes. This generates soluble ligands that chronically engage NKG2D on NK and CD8 T cells. Chronic engagement can downregulate NKG2D. This leads to immune-cell desensitization and immune escape [162,165,167,168,170,171].

EBV-encoded BART miRNAs further contribute to NK-cell evasion in EBV^+^ NPC. miR-BART7 suppresses the TGFβ1/c-Myc/MICA regulatory axis, thereby reducing surface MICA expression and weakening NKG2D-dependent NK-cell recognition. Loss-of-function studies show that miR-BART7 inhibition restores MICA expression, enhances NK-cell cytotoxicity, and reduces NPC growth in vivo with increased NK-cell infiltration. Conversely, forced BART7 expression downregulates MICA and promotes additional tumor-supportive features, including radioresistance and a stem-like phenotype [169]. Broader analyses suggest that BART miRNAs coordinately suppress NKG2D ligands and pro-apoptotic molecules such as PUMA, creating a microRNA-based shield against NK- and T-cell attack [169,172].

Chronic interferon signaling and PD-1/PD-L1 activation further dampen NK function. IFN-β, chemotherapy and radiotherapy upregulate PD-L1 on NPC cells and PD-1 on NK cells, reducing NK-mediated lysis; PD-1/PD-L1 blockade with nivolumab restores and can enhance NK cytotoxicity, particularly when combined with chemotherapy or radiotherapy [173,174].

Together, B7-H3 upregulation, modulation and shedding of NKG2D ligands, BART miRNA-mediated repression of MICA and pro-apoptotic targets, and interferon-driven PD-1/PD-L1 signaling create a virus-shaped immune evasion network in EBV-positive NPC [162,165,167,169,170,171,172,173,174].

### NPC-Specific Constraints on NK Recognition, Trafficking and Persistence

Critically, NPC is not simply a generic solid-tumor setting for NK therapy but a virus-shaped immune ecosystem in which EBV latent programs and the stromal/metabolic tumor microenvironment jointly reduce NK-cell susceptibility and access. Beyond the BART-miRNA effects summarized above, EBV latent proteins also remodel activating-ligand display. LMP2A can reduce the NKG2D ligands MICA and ULBP4 on EBV-infected cells. LMP1-associated signaling also reshapes the NPC microenvironment. This occurs through NF-kappaB, glycolytic and inflammatory programs. These programs favor suppressive stromal and myeloid circuits rather than durable NK activation [175,176,177,178]. In parallel, NPC samples show recurrent HLA-E/CD94-axis engagement, making the NKG2A checkpoint particularly relevant even when classical HLA-I expression is heterogeneous or partially reduced [175,177].

These receptor-level constraints are reinforced by soluble and metabolic suppression. TGF-β, IL-10, adenosine, hypoxia and lactate are recognized components of the NPC milieu. They impair NK-cell proliferation, degranulation, IFN-γ production and metabolic fitness. As a result, they limit the activity and persistence of both endogenous and adoptively transferred NK cells [156,159,177]. At the tissue level, fibroblast-rich stroma, galectin-rich suppressive networks and protease-driven remodeling further decrease effective NK engagement. Galectin-9-associated interactions have been implicated in NPC immune dysfunction. Matrix-remodeling enzymes and sheddases can also promote extracellular-matrix exclusion. They may also cause loss of membrane NKG2D ligands through soluble or exosomal release [156,157,166,177].

Trafficking is similarly likely to be rate-limiting. NPC lesions are rich in inflammatory chemokine gradients, yet the NK subsets best equipped for tissue entry and retention, and the receptors needed for efficient homing or retention, including CXCR3, CCR5 and CXCR6, may not be optimally represented or maintained after ex vivo expansion. Thus, future NPC-directed NK platforms will probably need not only stronger killing programs but also trafficking-aware design, for example chemokine-receptor engineering or combinations that normalize stromal and metabolic barriers [156,159,179].

## 6. NK-Cell-Based Therapies in Nasopharyngeal Carcinoma

### 6.1. Cetuximab Plus NK Cells: Proof-of-Concept for ADCC

Most non-keratinizing NPCs overexpress EGFR, supporting the use of anti-EGFR antibodies such as cetuximab in locoregionally advanced and recurrent/metastatic disease [167,179,180]. As an IgG1 antibody, cetuximab not only blocks EGFR signaling but also engages CD16 on NK cells to trigger ADCC. Preclinical models show that cetuximab-coated NPC and HNSCC cells induce robust NK degranulation and IFN-γ production in a CD16-dependent manner [181,182,183].

A phase I trial by Lim et al. combined standard-dose cetuximab with autologous ex vivo-expanded NK cells in heavily pretreated recurrent/metastatic NPC [183]. NK cells were expanded using K562-mb15-41BBL feeder cells, generating activated CD56dim CD16bright NK populations. The combination was well tolerated, with no dose-limiting toxicities or CRS, and did not exacerbate typical cetuximab toxicities. Four of seven patients achieved stable disease, and three patients who received two NK infusions had prolonged disease control (12, 13 and 19 months) despite multiple prior therapies [184]. Correlative data showed increased circulating activated NK cells and enhanced cetuximab-dependent ADCC after NK infusion [184].

Mechanistically, EBV promotes CD55 upregulation through LMP1-dependent activation of the NF-κB pathway. Increased CD55 expression reduces cetuximab sensitivity by blunting cetuximab-triggered ADCC and limiting NK-cell-mediated cytotoxicity, whereas CD55 knockdown restores NK-cell function and improves cetuximab efficacy in vitro and in NPC xenograft models [185]. These data support the concept that augmenting NK effector function and modulating complement regulation can improve the clinical impact of EGFR-targeted therapy in NPC [181,182,183,184,185].

### 6.2. Checkpoint Blockade, Radiotherapy and NK Cells in NPC

PD-1/PD-L1 blockade has become a standard component of first-line therapy for recurrent/metastatic NPC when combined with platinum-based chemotherapy, as shown in phase III trials such as JUPITER-02 (toripalimab), CAPTAIN-1st (camrelizumab) and RATIONALE-309 (tislelizumab) [186,187]. While initially viewed mainly as T-cell-directed therapies, PD-1/PD-L1 inhibitors also act on NK cells in NPC.

Using EBV-positive NPC cell lines and primary NK cells, Makowska et al. showed that IFN-β or cisplatin/5-FU upregulates PD-L1 on tumor cells and PD-1 on NK cells, leading to reduced degranulation and killing; nivolumab restored and sometimes enhanced NK cytotoxicity particularly in combination with chemotherapy [173]. Fractionated radiotherapy increased PD-L1, and death receptor expression on NPC cells, improving immunogenicity but also promoting PD-1/PD-L1-mediated resistance to NK attack; combined radiotherapy and PD-1/PD-L1 blockade significantly boosted NK-mediated killing of irradiated NPC targets [174,181].

Immunoprofiling of NPC biopsies shows that tumor-infiltrating NK cells often express PD-1 and other checkpoints (e.g., TIGIT, NKG2A) and exhibit exhausted transcriptional states in PD-L1-high tumors [185,188]. These findings support viewing PD-1/PD-L1 inhibitors in NPC as agents that reinvigorate both T and NK compartments, especially when combined with chemo- and radiotherapy that increase tumor immunogenicity but also upregulate PD-1/PD-L1 [16,171,173,175].

### 6.3. Targeting B7-H3 and NK Engagers in Head and Neck Cancer

As mentioned in Section 5, B7-H3 is EBV-inducible in NPC and broadly overexpressed in HNSCC, where it functions as an immune checkpoint. In EBV^+^ NPC, B7-H3 is highly expressed on tumor cells, correlates with reduced NK infiltration and poor prognosis, and its knockdown or blockade restores NK cytotoxicity and reduces tumor growth and metastasis [167,168]. Multi-omics profiling in HNSCC likewise identifies B7-H3 as an immunoregulatory, tumor-associated molecule [61,75].

B7-H3 is highly expressed in HNSCC, particularly in HPV-negative disease, making it an attractive target for NK-cell redirection. B7-H3-directed NK engagers have therefore attracted interest. The B7-H3 TriKE cam1615B7H3 retains the CD16/IL-15 activating backbone of the CD16/IL-15/CD33 TriKE but replaces the CD33-targeting domain with a B7-H3-specific binding moiety. It consists of a CD16-binding VHH, wild-type IL-15 and an anti-B7-H3 scFv, thereby redirecting NK cells toward B7-H3^+^ tumor cells. In preclinical models, cam1615B7H3 potently activated NK cells, promoted NK-cell proliferation and mediated strong B7-H3-specific tumor killing, with superior in vivo efficacy compared with IL-15 alone or B7-H3 bispecific controls. Its retained activity in hypoxic and 3D spheroid models further supports its relevance for the solid-tumor microenvironment [33,34,60,68].

CD16A-based engagers such as AFM24 (EGFR/CD16A) further validate the concept of redirecting innate immune cells in EGFR^+^ solid tumors. AFM24 has shown manageable safety and signs of activity in advanced EGFR-expressing cancers, including HNSCC [7,63,69].

More specifically, first-in-human AFM24 monotherapy data established a recommended phase 2 dose of 480 mg but showed mainly stable disease rather than frequent objective responses, again supporting combination-based development in EGFR-positive solid tumors [135].

Together, Section 6.1, Section 6.2 and Section 6.3 support combining B7-H3-targeted agents (antibodies, BiKEs/TriKEs, CAR-NK) with PD-1/PD-L1 inhibitors and radiotherapy in EBV^+^ NPC. In such combinations, B7-H3 blockade lifts a tumor-intrinsic NK checkpoint, TriKE-delivered IL-15 expands and supports NK cells even in hypoxic niches, and PD-1/PD-L1 blockade plus radiotherapy further enhance T- and NK-cell recognition [167,170,187,188,189,190,191,192].

### 6.4. Allogeneic NK and CAR-NK Approaches in NPC

Allogeneic NK- and CAR-NK cell platforms are particularly attractive for nasopharyngeal carcinoma (NPC) because they are available as off-the-shelf products, are generated independently of EBV-infected patient material, and can be readily integrated with standard treatment. In this context, NK-92, a continuously expanding allogeneic NK-cell line, has demonstrated robust cytotoxicity against both EBV-positive C666-1 cells and EBV-negative NPC cell lines, as well as against patient-derived xenograft (PDX) cells. Moreover, increasing NK-92-to-target cell ratios progressively reduced tumor-cell viability, with PDX-derived NPC cells appearing particularly sensitive [191].

Umbilical cord blood CD34^+^ HSC-derived NK cells engineered with PD L1 CARs (CAR pNK = CAR-engineered primary NK cells) efficiently kill EBV^+^ NPC cell lines and PDX derived tumor cells, induce degranulation and IFN γ secretion, and suppress tumor growth in humanized mouse models. PD L1 was selected as the CAR target because EBV-positive NPC commonly overexpresses this ligand, enabling CAR engineered NK cells to selectively recognize and eliminate tumor cells while overcoming the immune suppressive PD 1/PD L1 that normally inhibits NK cell function. When combined with nivolumab, CAR pNK cells produce synergistic antitumor effects and broaden activation of antigen presentation pathways, demonstrating that allogeneic CAR NK cells can reprogram EBV driven NPC immunity [141,142].

Cord blood-, HSC- and iPSC-derived NK platforms are already being tested in other cancers and are amenable to engineering (CARs, non-cleavable CD16, cytokine armoring) and cryopreservation, making them ideal candidates for NPC-specific off-the-shelf products to be combined with chemoradiotherapy and PD-1 blockade [193,194]. Antigen platforms developed in HNSCC, such as EGFR-targeted CAR-NK-92, are directly translatable to EGFR^+^ NPC [195], and PD-L1 CAR-NK regimens combined with pembrolizumab and N-803 (e.g., NCT04847466) in gastrointestinal and head and neck cancers provide a template for similar multimodal approaches in NPC [195,196].

Beyond salvage settings, NK therapy is moving earlier in the NPC treatment pathway. Apart from the cetuximab + autologous NK trial by Lim et al. [183], an adjuvant phase 1/2 study (AlloNK1, NCT07247201) now evaluates allogeneic NK infusions after chemoradiotherapy in high-risk locally advanced NPC, aiming to eradicate minimal residual disease and micrometastases [197]. Taken together, preclinical and early clinical data support the integration of standardized allogeneic NK and CAR-NK products into the current NPC paradigm alongside chemoradiotherapy and PD-1 blockade [193,194,195,197,198].

### 6.5. Critical Comparison with T-Cell-Based Approaches in NPC

Clinically, the established immunotherapy benchmark in recurrent/metastatic NPC is PD-1 blockade combined with chemotherapy [186,187]. In contrast, NK-directed clinical evidence in NPC remains early and largely proof-of-concept. The clearest direct clinical ADCC signal comes from a small phase I study combining cetuximab with adoptively transferred NK cells [184]. This distinction matters: T-cell-based approaches currently have stronger efficacy data in NPC, but they also depend more heavily on stable antigen presentation and intact HLA-restricted recognition, which EBV-driven tumors can erode through HLA dysregulation, epigenetic adaptation and antigenic heterogeneity [175,176]. By contrast, NK cells can exploit stress ligands, antibody opsonization and partial HLA-I loss, making them conceptually attractive for tumors that escape classical T-cell surveillance.

However, NK and T-cell platforms likely solve different problems rather than competing head-to-head. EBV-specific cytotoxic T-cell products can mediate durable disease control in selected NPC patients and offer true viral-antigen specificity [199,200,201,202,203], but they require individualized manufacturing, adequate T-cell fitness and persistence after infusion. NK products are more amenable to standardized allogeneic manufacturing and repeated off-the-shelf dosing, and they generally carry lower risks of CRS, ICANS and GVHD than allogeneic or heavily activated T-cell therapies; the trade-off is shorter persistence and, so far, less mature efficacy data in NPC [193,194,195,196,197,198,199,200,201,202].

A balanced translational view is therefore needed. PD-1 inhibitors and EBV-specific T cells currently define the clinically validated T-cell axis in NPC. In contrast, NK-based therapy may be most valuable as a complementary platform. NK-based approaches could reinforce cetuximab-like ADCC. They could also attack HLA-perturbed or antigen-escape variants. In addition, they may provide safer and more scalable cellular products. Such products could be combined with radiotherapy, checkpoint blockade and EBV-directed vaccination. In NPC, the strategic question is not NK versus T cells, but which compartment is being rescued at each stage of disease, and which escape mechanism—antigen loss, HLA restriction, stromal exclusion or metabolic paralysis—is dominant in a given patient [184,186,187,195,199,200,201,202,203,204,205].

## 7. EBV-Directed Strategies and NK Cells

In endemic regions, NPC is almost uniformly EBV-positive, making the viral genome an attractive set of tumor-selective targets that intersect with NK biology. EBV miR-BART7-3p is highly expressed in EBV^+^ NPC, promotes EMT, metastasis and PTEN/PI3K–Akt activation and enhances tumorigenicity in vivo; systemic administration of AuNP loaded with anti-EBV-miR-BART7-3p nanoparticles suppressed the growth of EBV-positive NPC xenografts, supporting BART7 as a therapeutically actionable target. Mechanistically, these gold nanoparticles were loaded with anti-BART7 molecules to selectively inhibit the viral microRNA oncogene EBV miR-BART7-3p in EBV-positive tumor cells [198]. BART7 and related miRNAs also modulate radiation sensitivity and NKG2D–MICA/B pathways, contributing to both stemness/radioresistance and NK evasion, and providing a mechanistic rationale for combining NK-based strategies with anti-BART therapeutics [197,204,205,206].

EBV latency antigen-directed vaccines offer a complementary approach. Recombinant MVA-EL (EBNA1/LMP2) and dendritic cell- or adenovirus-based LMP2 vaccines have shown safety and the induction of robust EBV-specific CD4^+^ and CD8^+^ T-cell responses with signals of disease control, particularly in patients with minimal residual disease [206]. Newer lipid-based LMP2 mRNA vaccines similarly elicit strong EBV-specific immunity and inhibit tumor growth in NPC models. Notably, in humanized mice bearing EBV^+^ NPC xenografts, combining an EBV mRNA vaccine with adoptive NK-cell transfer synergistically improved tumor control, leading to durable suppression or eradication and enhanced intratumoral NK activation compared with either modality alone [205].

These findings support a model in which EBV-directed strategies and NK-cell therapies are mutually reinforcing. Anti-BART7 or other miRNA-targeted approaches may restore NK-cell recognition of EBV^+^ NPC cells by increasing stress-ligand expression and could be most relevant in the setting of minimal residual disease rather than bulky tumor debulking. In parallel, EBV vaccines can expand virus-specific T-cell responses and may also promote NK-biased immune responses. Together, these strategies may create a more favorable antigenic and immunologic context for adoptive NK or CAR-NK products.

Minimal residual disease after chemoradiotherapy may be an especially suitable setting for such EBV-centered combinations, where vaccine-driven and NK-mediated immune pressure could eradicate residual EBV-driven clones and prevent relapse [197,204,205,206].

## 8. Conclusions

Overall, this review positions nasopharyngeal carcinoma as a particularly instructive model for translating NK-cell biology into clinically effective regimens. Rather than relying on NK-cell approaches as stand-alone therapies, the most credible path forward is rational combination: pairing NK-directed agents (including B7-H3-targeted antibodies or TriKE/CAR-NK platforms) with PD-1/PD-L1 blockade and radiotherapy to relieve NK checkpoints, sustain NK fitness, and broaden coordinated T- and NK-mediated tumor recognition. Off-the-shelf allogeneic NK and CAR-NK products appear especially compatible with NPC because they can be integrated into existing standards of care, and current directions are already moving beyond salvage treatment toward earlier-line or adjuvant strategies aimed at eliminating minimal residual disease after chemoradiotherapy. Finally, EBV-specific interventions provide a unique opportunity to “shape” the target. miRNA-directed approaches, such as anti-BART7, may resensitize EBV^+^ NPC to NK-cell attack. EBV vaccines can also supply focused immune pressure. This immune pressure may synergize with adoptive NK or CAR-NK therapy. Together, these strategies make the MRD setting a rational niche for EBV-centered, NK-enabled immunotherapy. Such an approach could help prevent relapse.

## Figures and Tables

**Table 1:** Major activating and inhibitory NK-cell receptors, their main ligands and functional roles.

Receptor/Family	Type	Main Ligands/Binding Partners	Main Function/Role
KIRs (e.g., KIR2DL, KIR3DL)	inhibitory	Classical HLA class I molecules (HLA-A, -B, -C)	Sense self–MHC I and deliver dominant inhibitory signals to prevent killing of healthy self-cells.
NKG2A/CD94	inhibitory	HLA-E molecules	Inhibits NK activation when HLA-E is expressed, contributing to tolerance to normal tissues.
LIR-1/ILT2 (LILRB1)	inhibitory	Classical and non-classical HLA class I molecules	Broad inhibitory receptor that dampens NK activation upon recognition of MHC I on target cells.
NKR-P1A/CD161	inhibitory	LLT1/CLEC2D	Tumor LLT1 inhibits NK-cell function and supports immune escape.
TIGIT	inhibitory	PVR (CD155), NECTIN2 (CD112)	Competes with DNAM-1 for shared ligands and transmits inhibitory signals, promoting NK exhaustion.
PD-1	inhibitory	PD-L1 (B7-H1), PD-L2	Immune checkpoint: on human NK cells, expression is context-dependent and often low/inducible rather than constitutive. In PD-1^+^ NK cells, engagement by PD-L1-expressing tumor or immune cells may contribute to reduced cytotoxicity and cytokine production.
TIM-3	inhibitory	Galectin-9, Phosphatidylserine, CEACAM1 (context-dependent)	In tumors persistent TIM-3 expression is commonly associated with dysfunctional or exhausted NK-cell states and reduced antitumor activity
LAG-3	inhibitory	MHC class II molecules	Coinhibitory receptor; reported on activated NK-cell subsets in some settings, such as cytokine-activated NK cells and CMV/NKG2C-driven adaptive NK-cell subsets, but its expression and inhibitory function on NK cells are less broadly established than in T cells.
CD96	inhibitory	PVR (CD155)	Negative regulator that counterbalances DNAM-1-mediated activation and limits NK-mediated cytotoxicity.
2B4 (CD244)	SAP-dependent co-regulatory (SAP = SLAM-associated protein)	CD48	In SAP-sufficient NK cells: usually co-stimulatory/activating signals; in SAP deficiency and some chronic stimulation settings: inhibitory.
NKG2D	activating	Stress-induced ligands: MICA/B, ULBP1–6	Recognizes ligands upregulated on virus-infected and malignant cells and triggers potent cytotoxicity.
DNAM-1 (CD226)	activating	PVR (CD155), NECTIN2 (CD112)	Promotes adhesion, immune synapse formation and killing of transformed or stressed target cells.
Natural cytotoxicity receptors (NCRs): NKp30, NKp44, NKp46	activating	Diverse ligands Including B7-H6, BAT3, heparan sulfate proteoglycans; viral hemagglutinins	Key triggering receptors for recognition of tumor and virus-infected cells; cooperate with others activating receptors.
CD16 (FcγRIIIa)	activating	Fc portion of IgG1 antibodies bound to target antigens	Mediates antibody-dependent cellular cytotoxicity (ADCC) against antibody-coated tumor cells.
NKG2C/CD94	activating	HLA-E molecules	Activating counterpart of NKG2A; contributes to expansion of adaptive/memory-like NK-cell subsets.
NKG2E/CD94	putative activating	HLA-E molecules (putative/less well-defined than with NKG2C)	CD94-associated receptor with unclear surface expression and function in NK cells; no broadly established activating role.
KIRs (e.g., KIR2DS1, KIR2DS4; human activating KIRs)	activating	Selected HLA class I allotypes in a peptide-selective, receptor-specific manner (best defined for KIR2DS1 with some C2+ HLA-C).	Provide activating input in selected allogeneic/inflammatory contexts; specificity is narrower and less certain than for inhibitory KIRs.
NKp80	activating	AICL (activation- induced C-type lectin) on myeloid cells	Co-stimulatory receptor that supports NK activation against stressed myeloid and tumor cells.
CD27 (subset/species-restricted), CD28 (rare on NK cells)	co-stimulatory	CD70 (for CD27), CD80/CD86 (for CD28, when expressed)	Provide additional activation and survival signals to NK subsets; CD27 biology is best established in mouse NK maturation and in some human subsets, whereas CD28 is not a general NK marker.
4-1BB (CD137), OX40 (CD134, less consistently reported on NK cells)	co-stimulatory	4-1BBL, OX40L on antigen-presenting or tumor cells	4-1BB is inducible on activated NK cells and can enhance effector function; OX40 expression/function on NK cells is context-dependent such as B-cell lymphoma models or exhausted/tumor-infiltrating NK-cell subsets in cervical cancer, but its expression and function on NK cells are less broadly established.
SLAMF7 (CS1/CRAAC)	activating/self-ligand	SLAMF7 (homotypic interaction)	NK-cell activation and ADCC against SLAMF7-expressing targets; therapeutic target in multiple myeloma with reduced off-tumor toxicity.

**Table 2:** Cytokine-based activation of endogenous NK cells.

Cytokine/Agent	Type/Main Receptor Engagement	Main Effects on NK Cells	Key Clinical/Functional Notes
High-dose IL-2	Common γ-chain cytokine; signals via trimeric IL-2R (CD25/CD122/CD132) on NK cells, CD8 T cells and Tregs	Expands and activates NK cells and effector CD8 T cells; enhances cytotoxicity and cytokine production	Induces durable complete responses in a small subset of patients with metastatic melanoma and renal cell carcinoma; activates/expands NK and effector CD8 T cells (antitumor effect); also expands CD25^high^ FOXP3^+^ Tregs; causes significant systemic toxicity (e.g., capillary-leak/vascular-leakage syndrome), limiting use to selected fit patients.
Recombinant IL-15	Common γ-chain cytokine; signals via IL-15Rα/IL-2Rβ/γc complex (trans-presentation) on NK cells and memory CD8 T cells	Promotes proliferation, survival and activation of NK cells and memory CD8 T cells without directly stimulating CD25high Tregs	Demonstrates robust NK and CD8 T-cell expansion in early-phase studies but generally limited clinical responses as monotherapy; serves as a foundation for next-generation IL-15 agonists.
N-803 (ALT-803)	IL-15 superagonist complex (IL-15 mutant bound to IL-15Rα sushi domain fused to IgG)	Potently expands and activates NK cells and CD8 T cells with relatively minor effects on Treg numbers; Enhances NK degranulation and IFN-γ production	In post-transplant hematologic malignancies and solid tumors, shows objective responses and strong NK activation; intravesical N-803 + BCG induce high complete-response rates and durable bladder-preserving control in BCG-unresponsive non-muscle-invasive bladder cancer, leading to regulatory approval in this setting.
Nanrilkefusp alfa (SOT101)	IL-2/IL-15 receptor βγ complex (CD122/IL-2/IL-15Rβ and CD132/common γ-chain, γc)	Dose-dependent expansion and activation of circulating and intratumoral NK cells and CD8 T cells	Early-phase studies in advanced solid tumors show biological activity with NK/CD8 expansion but predominantly stable disease or short-lived responses as monotherapy; being explored mainly in combination regimens.
Brief IL-12/IL-15/IL-18 pre-activation (cytokine-induced memory-like-CIML NK platform)	Ex vivo cytokine priming via IL-12R, IL-15R and IL-18R before adoptive transfer	Induces a memory-like NK-cell state with enhanced IFN-γ production, cytotoxicity, metabolic fitness and in vivo persistence	Used as an adoptive NK-cell platform rather than soluble cytokine monotherapy; clinical data support activity in AML, and more recent head and neck studies combining CIML NK cells with N-803 and ipilimumab confirm feasibility and biologic activity, although efficacy remains preliminary [40,41,42,43,44,45].
IL-2/IL-15–family cytokines (class effect)	Common γ-chain cytokines engaging IL-2R and IL-15R complexes	Robustly expand and activate endogenous NK-cell compartments, increase cytotoxicity and cytokine secretion	As single agents, rarely achieve durable tumor control; current development focuses on combinations with checkpoint inhibitors, therapeutic antibodies or intravesical BCG, and on incorporation into engager and cell-therapy platforms (e.g., TriKEs, IL-15-armored NK/CAR-NK).

## Data Availability

No new data were created or analyzed in this study. Data sharing is not applicable to this article.

## Abbreviations

The following abbreviations are used in this manuscript:

Abbreviation	Full Term
ADCC	antibody-dependent cellular cytotoxicity
AFM13	CD30/CD16A bispecific tetravalent TandAb antibody
ALT-803	IL-15 superagonist N-803
AML	acute myeloid leukemia
APRIL	a proliferation-inducing ligand, TNFSF13
BAFF	B-cell activating factor, also known as TNFSF13B
B-ALL	B-cell acute lymphoblastic leukemia
BCMA-targeted CAR-NK	B-cell maturation antigen-targeted CAR-NK cells
BiKEs/TriKEs	bispecific and trispecific NK-cell engagers
B-NHL	B-cell non-Hodgkin lymphoma
CAR-NK	chimeric antigen receptors NK cells
CIML NK cells	cytokine-induced memory-like NK cells
CLL	chronic lymphocytic leukemia
CRS	cytokine release syndrome
EBV	Epstein–Barr virus
ECM	extracellular matrix
EGFR	estimated glomerular filtration rate
FASL	FAS ligand
FCGR3A-158V/V alleles	Fc-gamma-receptors IIIa homozygous valine at position 158
FT500	off-the-shelf, iPSC-derived natural killer (NK) cell product designed to enhance responses to immune checkpoint inhibitors (ICI) and overcome ICI resistance in solid tumors
FT538	next-generation NK-cell product in Fate’s off-the-shelf iPSC pipeline containing enhanced features such as improved cytotoxicity, engineered persistence, enhanced antibody-dependent cell killing
FT596	first-in-class, multi-antigen-targeted, off-the-shelf, iPSC-derived CAR-NK cell therapy designed to treat B-cell lymphomas and leukemias
FT576	BCMA-targeted off-the-shelf iPSC-derived CAR-NK cell product incorporating a CAR, hnCD16 and IL-15 receptor fusion construct
GTB-3550	first-generation trispecific NK-cell engager (TriKE^®^)
GTB-3650	next-generation, camelid single-domain antibody-based TriKE^®^
GVHD	graft-versus-host disease
HLA	human leukocyte antigen
HNSCC	head and neck squamous cell carcinoma
HPV	human papillomavirus
HSC-derived NK platforms	hematopoietic stem cell-derived NK platforms
ICAN	immune effector cell-associated neurotoxicity syndrome
IFN-β	interferon-beta
IFN-γ	iterferon-gamma
IL-15	interleukin-15
IL-18	interleukin-18
IL-2	interleukin-2
iPSCs	induced pluripotent stem cells
MDS	myelodysplastic syndromes
MDSC	myeloid-derived suppressor cell
MHC class I	major histocompatibility complex class I
MRD	minimal residual disease
NK cells	natural killer cells
NKX101	allogeneic NKG2D-ligand-targeted CAR-NK product evaluated clinically in AML/MDS
NPC	nasopharyngeal carcinoma
NSG xenograft mouse	non-obese diabetic severe combined immunodeficiency interleukin-2 receptor gamma chain null mouse
PBMC	peripheral blood mononuclear cells
PDX cells	patient-derived xenograft cells
PGE_2_	prostaglandina E2
SOT101	IL-15 superagonist nanrilkefusp alfa
TGF-β	transforming growth factor-beta
TME	tumor microenvironment
TRAIL	tumor necrosis factor-related apoptosis-inducing ligand
